# Recent Advances in Nanotheranostic Agents for Tumor Microenvironment–Responsive Magnetic Resonance Imaging

**DOI:** 10.3389/fphar.2022.924131

**Published:** 2022-06-22

**Authors:** Longhai Jin, Chenyi Yang, Jianqiu Wang, Jiannan Li, Nannan Xu

**Affiliations:** ^1^ Department of Radiology, The Second Hospital of Jilin University, Changchun, China; ^2^ Department of Blood Transfusion, Central Hospital of Changchun, Changchun, China; ^3^ Department ofGeneral Surgery, The Second Hospital of Jilin University, Changchun, China

**Keywords:** nanomaterials, tumor microenvironment, stimuli-responsive, MRI, diagnosis of cancer

## Abstract

Nanomaterials integrating a variety of excellent properties (such as controllable/suitable size, surface modifier, and multifunctionality) have attracted increasing attention in the biomedical field and have been considered a new generation of magnetic resonance imaging (MRI) contrast agents (CAs). In recent years, stimuli-responsive nanomaterials with specifically responsive ability have been synthesized as MRI CAs, which can significantly improve the diagnostic sensitivity and accuracy depending on their outstanding performance. Furthermore, the inherent tumor microenvironment (TME) of malignant tumor is considered to possess several unique features, such as low extracellular pH, redox condition, hypoxia, and high interstitial pressure, that are significantly different from healthy tissues. Hence, constructing nanomaterials for TME-responsive MRI as an emerging strategy is expected to overcome the current obstacles to precise diagnosis. This review focuses on recent advances of nanomaterials in their application of TME-responsive MRI that trigger the diagnostic function in response to various endogenous stimulations, including pH, redox, enzyme, and hypoxia. Moreover, the future challenges and trends in the development of nanomaterials serving as TME-responsive MRI CAs are discussed.

## Introduction

Cancer has become a primary cause of death and a most serious threat to human health worldwide (Sung et al., 2021). Early and accurate diagnosis is crucial to conquering cancer, which can guide clinicians to design the most timely, suitable, and reasonable therapeutic strategies for cancer patients. Magnetic resonance imaging (MRI) as one of the most powerful non-invasive imaging instruments with advantages of non-radiation and excellent spatial resolution. It is widely used in the clinical diagnosis of cancer (Na et al., 2009). It can provide anatomical and functional information of organs and soft tissues, for instance, T1 MRI can display normal anatomy with excellent resolution and T2 MRI is adept at detecting tumors and inflammation lesions with outstanding soft tissue imaging. The introduction of contrast agents (CAs) further improves the sensitivity and capability of MRI to detect lesions, which offers unprecedented and comprehensive diagnostic information for clinicians (Lohrke et al., 2016). Gadolinium (Gd^3+^) chelate–based MRI CAs are most commonly used in clinics at present. However, these Gd-based CAs have their own limitations, such as very short internal circulation time, poor specificity, and relatively low relaxation efficiency. In order to overcome the above defects, it is necessary to use large doses of Gd-based CAs to increase the CA concentration in the target area (Raymond and Pierre, 2005). Afterward, high-dose injection of Gd^3+^ chelates may cause severe nephrogenic systemic fibrosis syndrome, as has been warned by the US Food and Drug Administration (FDA) (Penfield and Reilly, 2007; Perez-Rodriguez et al., 2009; Chen et al., 2012). Therefore, the comprehensive performance of currently available CAs in clinic is far from ideal. Research and development of innovative and efficient MRI CAs as a substitute for traditional Gd-based CAs is highly desirable and has become a very promising field.

Over the past decades, nanomaterials integrating a variety of excellent properties have been proposed as a new generation of CAs with great potential in MRI. As imaging CAs, nanomaterials have a series of attractive characteristics: 1) controllable and suitable size, which ensures sufficient circulation time in blood and clearance ability by kidneys or reticuloendothelial system (RES) (Longmire et al., 2011). 2) Surface modifiers, which allow them to be coated with biocompatible materials to improve their biocompatibility or conjugated with ligands to provide additional properties such as target-specific binding ability, barrier-penetrating ability, and long circulation time (Lim et al., 2015). 3) Multifunctionality, integrating multiple imaging modes into one platform, making them capable of providing comprehensive information for detecting lesions. The most frequently developed nanomaterials as T1 MRI CAs are Gd-based complexes (e.g., Gd_2_O_3_-, NaGdF_4_-, GdF_3_-, and Gd^3+^-doped nanoparticles [NPs]) (Liu et al., 2011; Kimura et al., 2012; Park et al., 2012; Passuello et al., 2012; Xing et al., 2014; Du et al., 2017) and paramagnetic manganese (Mn)-based composites (e.g., MnO NPs, MnO_2_ nanosheets, and Mn_3_O_4_ NPs) (Kim et al., 2011; Chen et al., 2014; Li et al., 2017), which can shorten their longitudinal relaxation time (T1) due to their excellent ability to interact with hydrogen protons in the surrounding water. Superparamagnetic iron oxide (SPIO) NPs are the other kind of popular CAs which can shorten transverse relaxation time (T2) because of their intrinsic magnetic properties, serving as T2 MRI CAs (Laurent et al., 2008; Tong et al., 2010). Despite the variety of nanomaterials with remarkable performance having been synthesized as MRI CAs which undeniably improved the sensitivity of detecting subtle lesions to a certain extent, there are still some shortages that cannot be neglected. For instance, 1) the majority of current MRI CAs possess the function of enhancing MR contrast signals “always on” regardless of their specific accumulation in target tumors or tissues, leading to poor target-to-background signal ratios. Therefore, it is difficult to distinguish the region of interest from surrounding normal tissues due to non-specific signal enhancement, which may lead to a false-positive diagnosis or missed diagnosis (Yoo and Pagel, 2008; Fu et al., 2021). 2) Dissatisfactory relaxivity causes insufficient sensitivity to detect early-stage tumors or tiny lesions, which may miss the optimal opportunity for treatment (Yu et al., 2014; Lee et al., 2015). 3) Inadequate internal circulation time results in narrow observation window of MRI, reduces diagnostic efficiency, and impedes their applications (Kielar et al., 2010; Ai, 2011; Shen et al., 2017).

Consequentially, designing stimuli-responsive CAs with specifically responsive ability is expected to overcome the above obstacles. Compared with traditional CAs, these stimuli-responsive CAs have exhibited conspicuously enhanced contrasts between target lesions (especially the small lesions or early-stage tumors) and normal tissues. Many endogenous or exogenous stimuli have been exploited to serve as triggers for stimuli-responsive CAs previously, including endo-stimuli (internal) such as pH, redox, and enzymes, and exo-stimuli (external) such as light, temperature, ultrasound, and electric or magnetic fields (Kang et al., 2017; García-Hevia et al., 2019). In addition, the tumor microenvironment (TME) of various types of malignant tumors, affected by malignant proliferation and metabolism, is considered to possess several unique features such as low extracellular pH, redox conditions, hypoxia, and high interstitial pressure (Danhier et al., 2010; Joyce and Fearon, 2015; Zhu et al., 2022). Nanomaterial-based TME-responsive CAs have attracted widespread attention for personalized cancer diagnosis owing to their outstanding performances in tumor-specific imaging, which could provide more accurate information for precise diagnosis of cancer and optimize treatment strategies (Yang et al., 2018; Wang J. et al., 2021).

**FIGURE 1:**
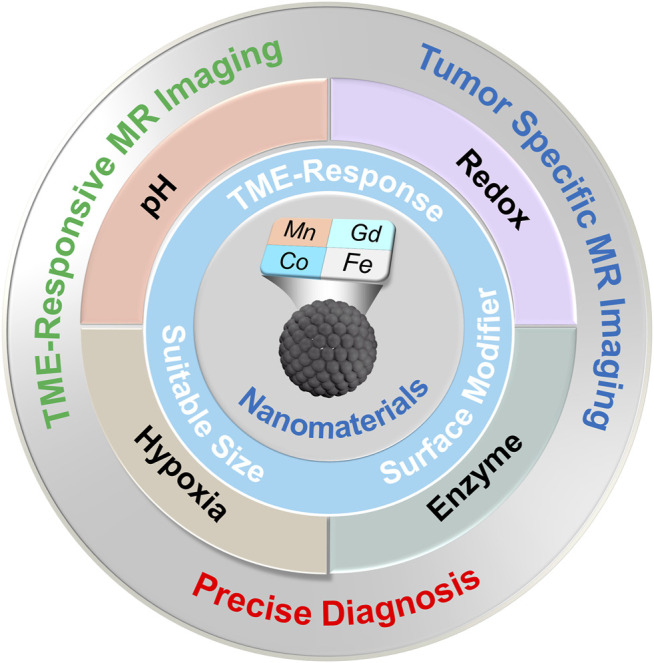
General strategies to synthesize nanotheranostic agents for TME-responsive MRI.

This review aims to summarize the recent advances and future prospects of nanomaterial-based CAs in their TME-responsive MRI applications rather than attempting to thoroughly contain the whole field (Figure 1). The inherent TME properties of tumors are significantly different from healthy normal tissues, which have been utilized in developing stimuli-responsive nano-MRI CAs for tumor-specific imaging. In this review, TME-responsive MRI CAs are classified according to types of endogenous stimulation, including pH, redox, enzyme, and hypoxia.

## pH-Responsive MRI Contrast Agents

According to the Warburg effect, abnormal and rapid proliferation of tumor cells consumes a lot of oxygen and nutrients, leading to acidosis and a reduced pH in the neighboring microenvironment (Vaupel et al., 1989; Webb et al., 2011). Thus, the extracellular pH values in TME are typically acidic in many tumors, differing significantly from those of normal tissues and blood (Lee et al., 2007). The pH-responsive MRI CAs provide noninvasive contrast enhancement depending on pH, which could be applied to tumor-specific imaging. General designs of pH-responsive MRI CAs can be classified by imaging components.

### Gd-Based pH-Responsive MRI CAs

Gd chelate is the most commonly used commercial MRI CA, so Gd-based pH-responsive MRI CAs have been widely studied. Zhu et al. attached pH-responsive block polymers on the surface of Gd-based NPs to achieve a surface modified Gd-metal organic frame (MOF) structure as a pH-responsive CA for MRI, which exhibited good performance in a series of characterization tests as well as imaging tests (Zhu et al., 2016). Its longitudinal relaxivity (r_1_) changed with the variation of environmental pH. The r_1_ value ranged from 6.6 mM^−1^ s^−1^ at pH 7.3 to 11.7 mM^−1^ s^−1^ at pH 6.6. To improve the biocompatibility and biodegradability of CAs, silkworm sericin (SS) was used to cross-link with Gd-based NPs by Huang et al. (Huang et al., 2021). A pH-responsive Gd-based NPs SS@GAH-GdCl_3_ with r_1_ values as 16.4 mM^−1^ s^−1^ at pH 5.8 and 9.2 mM^−1^ s^−1^ at pH 7.4 was obtained that showed a certain degree of increase with the decrease of pH value.

### Mn-Based pH-Responsive MRI CAs

As an essential element, manganese participates in many cellular processes *in vivo*, which are of great importance to growth, development, and cellular homeostasis of the normal body (Aschner and Aschner, 2005; Erikson et al., 2005). Apart from Mn ions playing a key role in physiological processes owing to their redox and catalytic nature, Mn also possesses significant magnetic properties. Thus, Mn-based NPs and CAs have entered the field of MR imaging diagnosis and received extensive attention. In spite of some Mn-based CAs serving as regular T1 CAs and having achieved similar effects as clinical Gd-based CAs, a novel class of Mn-based nanomaterials has been designed with improved imaging sensitivity and is potentially expected to be substitutes for traditional CAs. These nanomaterials were constructed to respond to biological microenvironments, mainly to pH and redox potential variations. Chen et al. introduced pH-sensitive degradable manganese oxide (MnO) NPs into mesopores of hollow silica nanocapsules (HMCNs) through in-site redox reaction, then gained MnO-HMCNs as a pH-responsive CA for T_1_ MRI (Chen et al., 2012). Under weak acidic conditions, MnO dissolves into Mn ions, causing a great increase in r_1_ value (8.81 mM^−1^s^−1^), which is 11 times higher than that in neutral conditions and 2 times higher than commercial Gd-based CAs. To improve the diagnostic specificity and sensitivity, stimuli-responsive multiple-mode MRI CAs are extremely advisable for obtaining overall and detailed diagnostic information to complement the deficiencies of each other. Our group synthetized cobalt phosphides (Co-P) as core, coating with manganese dioxide (MnO_2_) nanosheets to obtain Co-P@mSiO_2_–MnO_2_ for pH-responsive T1/T2 dual-mode MRI-guided synergistic anticancer therapy (Jin et al., 2017). For imaging, Co-P core was applied to T2 MRI owing to its intrinsic magnetic properties, and biodegradable MnO_2_ was employed as pH-responsive T1 MRI CA. Under acidic conditions, the r_1_ value of Co-P@mSiO_2_–MnO_2_ was determined to be 9.05 mM^−1^s^−1^, the r_2_ value was 253.44 mM^−1^s^−1^ which increased approximately 1.5 times than under neutral conditions.

### Fe-Based pH-Responsive MRI CAs

Fe is another essential element in the human body, mainly existing as ferritin in blood circulation. Paramagnetic Fe^3+^ is regarded as a promising candidate as T1 MR CAs owing to its large number of unpaired electrons (Wu et al., 2014; Zhang et al., 2017). Qu et al. recently fabricated an Fe-based biomimetic melanin-like multifunctional nanoagent (amino-Fe-PDANPs) as a positive pH-responsive MRI CA (Qu et al., 2021). Polydopamine was employed as a pH-responsive sensitizer for imaging. The r_1_ value was 10.0 mM^−1^s^−1^ at pH 7.5, then increasing to 15.4 mM^−1^s^−1^ at pH 6.5. It has been proven that the approachability of water molecules to the paramagnetic centers is the critical factor for T1 signal enhancement (Taylor et al., 2008). Interestingly, Fe-based NPs are also extensively applied as T2 MR Cas. Their magnetic property reduces rapidly with decreasing size owing to the reduction of magnetic anisotropy and spin disordering on the surface of NPs (Jun et al., 2008). Liu et al. synthesized PEGylated ultrasmall superparamagnetic iron oxide nanoparticles (USPIONs) by incorporating the methods of microemulsion and biomineralization, and coating with CaCO_3_ to obtain PEG-USPIONs@CaCO_3_ as a pH-responsive T2-T1–switchable MRI CA (Liu et al., 2021). The USPIONs agglomerated compactly inside the nanostructure, resulting in the enhancement of the T2 signal. While exposed to the acidic conditions in the tumor microenvironment, the CaCO_3_-layer releases free USPIONs by degradation, then switching to T1 MRI signal enhancement. With the gradual decrease of pH value, the r_2_/r_1_ ratio reduces substantially. In brief, PEG-USPIONs@CaCO_3_ will change from T2 CA in the neutral pH condition to T1 CA in the acidic environment of tumors. Not coincidentally, He et al. reported an extremely small iron oxide nanoparticle (ESIONP)–based pH-responsive T1-T2–switchable MRI CA which exhibited good performance (He et al., 2020).

## Redox-Responsive MRI Contrast Agents

In addition to weakly acidic pH, redox state as another characteristic biomarker of TME has drawn remarkable attention. Glutathione (GSH), among a variety of redox couples, is usually considered the most important thiol-disulfide redox buffer in charge of maintaining the balance of intracellular redox reactions (Do et al., 2014; Zhao et al., 2022). The concentration of GSH in tumor cells is much higher than that in normal tissues or blood (Cheng et al., 2011). Therefore, reduction-sensitive disulfide bonds and hydrogen peroxide (H_2_O_2_)–responsive boronated moieties were extensively used as redox-responsive MRI CAs, While Gd itself cannot be redoxed, many other metal ions have been developed, such as Mn, Fe, Co, Eu, and Cu.

### Mn-Based Redox-Responsive MRI CAs

Recently, Wang et al. developed an intelligent redox-responsive nanoplatform (MUM NPs) via the coprecipitation process involving upconversion NPs (UCNPs) and aggregation-induced emission-active photosensitizers, as well as an *in situ* generation process of MnO_2_ as the outer shell (Wang Y. et al., 2021). MUM NPs exhibited high specificity to TME, rapidly exhausting intracellular GSH and efficiently generating Mn^2+^, which were instrumental in ROS preservation and T1 MRI enhancement, respectively. This nanoplatform performed well in redox-responsive MR imaging. In addition to single-mode stimuli-responsive MRI, Kim et al. designed a redox-responsive T1/T2 dual-mode MRI CA, integrating a superparamagnetic core (Fe_3_O_4_) and a paramagnetic shell (Mn_3_O_4_) into a core-shell structure through a seed-mediated growth process (Kim et al., 2016). Under the stimulation of tumors’ intracellular reducing environments by glutathione, the Mn_3_O_4_ shell will be decomposed into Mn^2+^ for T1 signal enhancement and allow Fe_3_O_4_ to interact with water protons for T2 signal enhancement. Under GSH-free conditions, the r_1_ was 2.4 mM^−1^s^−1^ and the r_2_ was 92.2 mM^−1^s^−1^. After activation in GSH solution, the r_1_ and r_2_ values increased to 16.1 and 258.6 mM^−1^s^−1^, illustrating that this nanoplatform can be qualified as redox-responsive T1/T2 dual-MRI CA.

### Fe-Based Redox-Responsive MRI CAs

The yolk-shell type of GSH-responsive nanovesicles (NVs) were synthesized to encapsulate USPIO NPs and chemotherapy drugs by Liu et al.; the obtained USD NVs can respond to GSH-releasing drugs and activate T1 signal enhancement (Liu et al., 2020). The r_1_ value increased obviously to 3.1 mM^−1^s^−1^ in the presence of GSH. For switchable MRI, Cao et al. encapsulated citric acid–modified ESIONPs-CA in disulfide cross-linked poly (CBMA) nanogels, further introducing tumor-targeted c (RGD) ligand to obtain ICNs-RGD (Cao et al., 2020). With the stimulation of GSH, ICNs-RGD are rapidly degraded, and the agglomerated ESIONPs are dispersed evenly, which achieves switching from a T2 CA to a T1 one. ICNs-RGD exhibits a T2 contrast effect (dark) during its transport in the vessel, then switches to a T1 contrast effect (bright) after reaching the tumor region with a redox microenvironment. After stimulation by GSH, the r_1_ value was increased mildly from 5.56 to 7.40 mM^−1^s^−1^ and the r_2_ value drastically decreased from 103.01 to 14.36 mM^−1^s^−1^, which demonstrated that ICNs-RGD had the potential to be an efficient MRI CA in clinics.

## Enzyme-Responsive MRI Contrast Agents

Because of their unique substrate specificity and high selectivity as well as efficient catalysis in biochemical reactions, enzymes play an indispensable role in most biological and metabolic processes, which can be associated with a series of pathological changes, such as tumors, inflammation, and so on (Zhang et al., 2014; Wang et al., 2019). In particular, cathepsin B and matrix metalloproteinases (MMPs) with elevated expression in the tumor environment participate in numerous biological processes associated with cancer, such as progression, metastasis, and angiogenesis. These tumor-associated enzymes can be regarded as stimulators for enzyme-responsive imaging or treatment of cancer (Hu et al., 2012). By means of one step reaction, Sun et al. synthesized hyperbranched poly (oligo-(ethylene glycol) methacrylate)-Gd complexes (HB-POEGMA-Gd and HB-POEGMA-cRGD-Gd), which employed lysosomal cathepsin B as a stimuli-response component to realize enzyme-responsive T1 CA (Sun et al., 2016). Their r_1_ values were 12.25 and 14.65 mM^−1^s^−1^, respectively. Recently, Yan et al. reported a Gd-based enzyme-responsive MRI and NIR fluorescence imaging CA (P-CyFF-Gd) with alkaline phosphatase (ALP) as a model enzyme (Yan et al., 2019). Upon ALP activation, r_1_ increased from 8.9 to 20.1 mM^−1^s^−1^, which demonstrated that P-CyFF-Gd can be qualified as a new generation of enzyme-responsive T1 CA. Another Gd-based enzyme-responsive T1 CA was designed through a self-assembly approach with caspase-3/7 as an activator by Ye et al. (Ye et al., 2014). The r_1_ value significantly increased from 10.2 mM^−1^s^−1^ before activation to 19.0 mM^−1^s^−1^ after activation. For enzyme-responsive T2 imaging, Gallo et al. constructed Fe-based MMP, enzyme-activatable, and tumor-specific targeting NPs, which were tethered with CXCR4-targeted peptide ligands for targeting tumors (Gallo et al., 2014). Upon MMP activation, the structure of NPs changes to that of a self-assembled superparamagnetic cluster network through a cycloaddition reaction, resulting in T2 signal enhancement.

## Hypoxia-Responsive MRI Contrast Agents

The malignant proliferation of tumors depletes a large amount of oxygen, resulting in the hypoxia of the tumor environment. As an inevitable feature of tumors, hypoxia is considered to be the obvious causative factor of therapeutic resistance and metastasis (Gatenby and Gillies, 2008). Therefore, exploiting novel nanotheranostics to achieve accurate diagnosis of hypoxia and timely treatment simultaneously has drawn great attention in biomedical research as well as clinical studies. The Gd complex of 1,4,7,10-tetraazacyclododecane-1,4,7,10-tetraacetic acid with a 2-nitroimidazole attached to one carboxyl group by an amide linkage was synthesized by Rojas-Quijano et al.; the r_1_ value was determined to be 6.38 mM^−1^s^−1^. The MR imaging results demonstrated that the nitroimidazole-derivant modified nanoprobe could be qualified as a hypoxia-responsive T1 CA to distinguish hypoxic from normoxic tissues. For Mn-based CAs, Song et al. constructed a rattle-structured NP consisting of a UCNP core, a hollow mesoporous silica shell, and hypoxia-sensitive MnO_2_ nanosheet modification (Song et al., 2018). MnO_2_ could be disintegrated into Mn^2+^ to achieve hypoxia-responsive T1 signal enhancement, and the r_1_ value is 1.137 mM^−1^s^−1^ after activation. O’Neill et al. studied the possibility of Co-based bioreductive pro-drugs working as a hypoxia-responsive MRI CA. Once in the hypoxic environment of tumors, the diamagnetic Co(III) ions of the CA will be reduced to ions of paramagnetic Co(II), ones which could significantly shorten the T2 relaxation to realize a T2 signal enhancement.

## Discussion

TME-responsive MRI CAs have been widely developed for accurate diagnosis of cancer because of their outstanding properties. The recent work on nanomaterials for TME-responsive MRI is summarized in Table 1. Although great efforts have been devoted to exploring a variety of TME-responsive MRI CAs in the biomedical field, there are still some problems that need to be solved for accelerating their clinical transformation. For instance, 1) up to date, most studies of stimuli-responsive CAs based on nanomaterials stay in the stage of concept verification as well as the research of laboratory application; systematic and in-depth researches are needed to promote their clinical application. 2) The safety concerns of these CAs need to be investigated more thoroughly, including their long-term biocompatibility, pharmacokinetics, biodistribution, biodegradability, and excretion, which are vitally important for their clinical transformation. 3) Despite numerous stimuli-responsive CAs exhibiting good performance, nanomaterials with better properties (such as high relaxivity, adequate circulation time, and appropriate stimuli-response function) should be explored to further improve the imaging effect and diagnostic efficiency. We believe that ingenious design and construction of TME-responsive MRI CAs will greatly promote the development of early and accurate cancer diagnosis in the future.

**TABLE 1:** Summary of recent work on nanomaterials for TME-responsive MRI.

Nanomaterial	Responsive	Imaging component	Imaging mode	Relaxivity		References
				Before activation	After activation	
GdNPs	pH	Gd	T1	r_1_ = 8.3	r_1_ = 11.7	Zhu et al. (2016)
Gd-PCNPs	pH	Gd	T1	r_1_ = 6.62	r_1_ = 10.01	Jiang et al. (2017)
SS@GAH-GdCl_3_	pH	Gd	T1	r_1_ = 9.2	r_1_ = 16.4	Huang et al. (2021)
HMCNs	pH	Mn	T1	r_1_ = 0.79	r_1_ = 8.81	Chen et al. (2012)
Mn-LDH	pH	Mn	T1	r_1_ = 1.16	r_1_ = 9.48	Li et al. (2017)
Co-P@mSiO_2_@DOX-MnO_2_	pH	Mn, Co	T1, T2	-	r_1_ = 9.05	Jin et al. (2017)
				r_2_ = 169.93	r_2_ = 253.44	
Amino-Fe-PDANPs	pH	Fe	T1	r_1_ = 10.0	r_1_ = 15.4	Qu et al. (2021)
ESIONP system	pH	Fe	T1 to T2	r_1_ = 5.71	r_1_ = 3.88	He et al. (2020)
				r_2_ = 9.11	r_2_ = 42.2	
PEG-USPIONs@CaCO_3_	pH	Fe	T2 to T1	-	-	Liu et al. (2021)
MUM NPs	Redox	Mn	T1	r_1_ = 0.12	r_1_ = 6.89	Wang et al. (2021b)
RANS	Redox	Mn	T1, T2	r_1_ = 2.4	r_1_ = 16.1	Kim et al. (2016)
				r_2_ = 92.2	r_2_ = 258.6	
USD NVs	Redox	Fe	T1	-	r_1_ = 3.1	Liu et al. (2020)
HIONPs	Redox	Fe	T1, T2	-	r_1_ = 41.3	Xu et al. (2021)
					r_2_ = 118.7	
ICNs-RGD	Redox	Fe	T2 to T1	r_1_ = 5.56	r_1_ = 7.4	Cao et al. (2020)
				r_2_ = 103.01	r_2_ = 14.36	
P-CyFF-Gd	Enzyme	Gd	T1	r_1_ = 8.9	r_1_ = 20.1	Yan et al. (2019)
HB-POEGMA-cRGD-Gd	Enzyme	Gd	T1	-	r_1_ = 14.65	Sun et al. (2016)
C-SNAM	Enzyme	Gd	T1	r_1_ = 10.2	r_1_ = 19.0	Ye et al. (2014)
IONPs	Enzyme	Fe	T2	-	-	Gallo et al. (2014)
Gd-complexes	Hypoxia	Gd	T1	-	r_1_ = 6.38	Rojas-Quijano et al. (2012)
DOX-UCHSM-PEG-DOTA	Hypoxia	Mn	T1	r_1_ = 0.112	r_1_ = 1.137	Song et al. (2018)
CoTPAx complexes	Hypoxia	Co	T2	-	-	O'Neill et al. (2017)
